# Physical Activity, Smoking, and Alcohol Consumption in Association with Incidence of Type 2 Diabetes among Middle-Aged and Elderly Chinese Men

**DOI:** 10.1371/journal.pone.0077919

**Published:** 2013-11-04

**Authors:** Liang Shi, Xiao-Ou Shu, Honglan Li, Hui Cai, Qiaolan Liu, Wei Zheng, Yong-Bing Xiang, Raquel Villegas

**Affiliations:** 1 Department of Medicine, Division of Epidemiology and Vanderbilt Epidemiology Center, Vanderbilt University Medical Center, Nashville, Tennessee, United States of America; 2 Department of Diabetes Control and Prevention, Shanghai Municipal Center for Disease Control and Prevention, Shanghai, People’s Republic of China; 3 Department of Epidemiology, Shanghai Cancer Institute, Shanghai, People’s Republic of China; 4 Huaxi School of Public Health, Sichuan University, Chengdu, People’s Republic of China; 5 Department of Medicine, Division of Diabetes, Endocrinology and Metabolism and Vanderbilt Epidemiology Center, Vanderbilt University Medical Center, Tennessee, United States of America; Federal University of Rio de Janeiro, Brazil

## Abstract

**Background:**

Type 2 diabetes mellitus (T2DM) is a prevalent chronic disease worldwide. The prevalence of T2DM is increasing rapidly in China. Understanding the contribution of modifiable lifestyle factors on T2DM risk is imperative to prevent the development of T2DM in China.

**Methods:**

We examined associations between lifestyle factors including physical activity, smoking and alcohol consumption with incidence of T2DM among middle-aged and elderly men in urban Shanghai. Information on socio-demographics, lifestyle habits, dietary habits, and disease history was collected via in-person interviews. Anthropometric measurements were taken. A total of 51 464 Chinese men aged 40–74 years free of T2DM, coronary heart disease (CHD), and stroke at baseline were included in the current study. Incident T2DM was identified through follow-up surveys conducted every 2–3 years. Cox proportional hazard analyses were conducted to evaluate associations between lifestyle risk factors and incidence of T2DM.

**Results:**

We documented 1304 new cases of T2DM during 276 929 person-years of follow-up (average: 5.4 years). Physical activity was inversely associated with T2DM risk. Daily living, commuting, and total physical activity METs had inverse negative dose-response relationships with T2DM (*P*-trend = 0.0033, 0.0022, and <0.0001, respectively). Regular participation in exercise or sports reduced T2DM risk (HR = 0.86, 95%CI: 0.76–0.98). Moderate alcohol intake (1–3 drinks/day) was inversely related to T2DM risk (HR = 0.80, 95%CI: 0.67–0.94). Cigarette smoking, on the other hand, was associated with increased T2DM risk; HRs were 1.25 (95%CI: 1.00–1.56) for smoking more than 20 cigarettes per day and 1.28 (95%CI: 1.04–1.57) for smoking more than 40 pack-years.

**Conclusions:**

Physical activity and moderate alcohol intake are inversely associated with T2DM risk, whereas smoking was positively associated with T2DM risk among middle-age and elderly Chinese men. Preventive measures should be developed to focus on these modifiable lifestyle habits to reduce the upward trend of T2DM.

## Introduction

Type 2 diabetes mellitus (T2DM) has become one of the most prevalent chronic diseases worldwide [1]–[3]. The prevalence of T2DM is increasing rapidly not only in developed countries but also in developing countries [4]. According to the China National Diabetes and Metabolic Disorders Study, in 2008, there were 92.4 million adults living with diabetes in China [5]. There is a need for effective strategies aimed at the primary prevention of this disease.

The importance of lifestyle factors in the etiology of T2DM has been well documented in Western populations [6]. However, limited data are available on the associations between lifestyle risk factors and T2DM in non-Western countries, including China. As the world's largest developing country, China has encountered enormous economic development in the past several decades, especially in large cities such as Shanghai. Many Chinese are facing a rapid transition to a more Westernized lifestyle. Understanding the contribution of lifestyle factors to T2DM risk is imperative to prevent the development of T2DM in China.

In 2002, we launched a large, population-based cohort study of men in Shanghai, China, the Shanghai Men’s Health Study (SMHS), with the main focus of investigating the impact of diet and other lifestyle factors on the risk of chronic diseases. In this population, the prevalence of smoking is high (65.27%) and the types of alcoholic beverages consumed differ substantially from Western populations [7]. We evaluated whether lifestyle factors associated with T2DM in Western countries are similarly related to the incidence of T2DM among Chinese men.

## Methods

### Study Population

The SMHS is an ongoing, population-based, prospective cohort study of 61 491 middle-aged and elderly men living in urban Shanghai. Study recruitment was conducted from April 2002 to June 2006. Description of the study design and baseline questionnaires have been previously published elsewhere [8], [9]. Briefly, a total of 61,491 men (response rate = 74.0%) aged 40–74 years living in 8 urban communities of Shanghai with no history of cancer at recruitment were enrolled. In-person interviews were conducted by trained interviewers (retired nurses or physicians), and information on socio-demographic characteristics, lifestyle factors (including physical activity, smoking habits, and alcohol consumption), dietary habits, and medical history was collected. Anthropometric measurements including weight, height, and circumferences of the waist and hips were taken according to a standard protocol at baseline. The Institutional Review Boards of Vanderbilt University Medical Center and the Shanghai Cancer Institute approved the study protocols. All participants provided written informed consent.

### Physical Activity Assessment

At baseline recruitment, information on physical activity was obtained using a validated physical activity questionnaire (PAQ) [10]. The questionnaire evaluated physical activity during the 5 years preceding the interview and covered physical activity related to leisure time activities (LPA), daily living activities (DPA), and commuting to/from work (CPA). For LPA, participants were asked whether they had engaged in regular exercise or sports (at least once a week for 3 months continuously) over the preceding 5 years. Exercisers were asked to report details for up to three types of exercise/sports (i.e., type, hours/week, and years of participation in each activity). For DPA, participants were asked about participation in daily activities (i.e., walking, stair climbing, bicycling, and household activities) and, for CPA transportation (i.e., walking and cycling to/from work, daily errands).

Energy expenditure in standard metabolic equivalent values (METs) was used to estimate the intensity of different types of physical activity [11]. Exercise/sports-related energy expenditure was estimated by the weighted average of energy expended in all activities reported during the 5 years preceding the interview (METs hours/week/year). Individual non-exercise–related activities were estimated using the following standard METs: housework, 2.0 METs; walking, 3.3 METs; stair climbing, 9.0 METs; and bicycling, 4.0 METs [11]. We calculated total physical activity (total METs) by combining energy expenditure from all types of physical activity (LPA, DPA, and CPA).

### Cigarette Smoking Assessment

Participants were asked whether they had ever smoked at least one cigarette per day for more than 6 months. For men who answered yes to this question, a detailed smoking history was taken, which included age when they started smoking, age when they stopped (for those who quit), and for current smokers, the number of cigarettes smoked per day. Pack-years (packs smoked per day multiplied by years of smoking) were calculated to estimate lifetime exposure to cigarette smoking.

### Alcohol Consumption Assessment

Participants who reported consuming alcohol on a regular basis (at least once per week) for more than 6 months were defined as drinkers and were asked for the age at which they started and stopped regular drinking. Current drinkers were also asked to provide information about the types, frequencies, and usual quantity of their alcohol intake (rice wine, grape wine, beer, and liquor separately). One unit of alcohol (1 drink) was defined as one 4-ounce glass of wine, one 12-ounce can of beer, or one ounce of liquor [12]. Total alcohol consumption was calculated by adding units of intake for all alcoholic beverages. We then classified participants as follows: non-drinkers, occasional or light drinkers (<1 drink/day), moderate drinkers (1.0–2.99 drinks/day), and heavy drinkers (≥3 drinks/day). We also examined associations between different alcoholic beverages and T2DM. Grape wine consumption was uncommon in this population and was combined with rice wine in the analyses.

### Dietary and other Exposure Assessments

Information on dietary intake was evaluated by a validated food frequency questionnaire [13] and total energy intake was estimated. Socio-demographic factors such as age, occupation (professional, clerical, manual), level of education (none/elementary school, middle school, high school, college), per capita monthly income (<500, 500–999, 1,000–1,999, >1,999 *yuan*), history of hypertension, and family history of diabetes were assessed at baseline and considered to be potential confounders in the analyses.

### Outcome Ascertainment

Every 2–3 years, in-person, follow-up surveys were conducted to record the occurrence of cancer and other chronic diseases, including T2DM, in the cohort. To date, two in-person surveys (2004 to 2008, 2008 to 2011) have been completed, with response rates of 97.7% and 91.9%, respectively. Participants were asked if they had been diagnosed with diabetes by a physician. Those who reported having T2DM were also asked about their blood glucose levels. We considered a case of T2DM to be “confirmed” if the participant’s reported glucose level met at least one of the American Diabetes Association (ADA) recommended T2DM blood glucose criteria: 1) fasting glucose level greater than or equal to 7 mmol/l on at least two separate occasions, 2) an oral glucose tolerance test (OGTT) performed in their doctor’s office with a value greater than or equal to 11.1 mmol/L, and/or 3) use of hypoglycemic medication (i.e., insulin or oral hypoglycemic drugs) (10). Other cases were considered to be “probable” cases. We performed analyses with all self-reported T2DM cases included, but after excluding the “probable” cases, we found similar trends. In this report, we present results that include all cases of self-reported T2DM.

### Statistical Methods

There were 51 464 SMHS participants who were free of T2DM and/or glycosuria and cardiovascular disease at baseline. Differences in socio-demographic characteristics and other risk factors by diabetes status were evaluated using a t-test for continuous variables and a chi-square test for categorical variables. Person-years for each participant were calculated as the interval between baseline to diagnosis of T2DM, death, or completion of the latest follow-up interview. Cox proportional hazard models were used to estimate associations between physical activity level, smoking, alcohol consumption, and incidence of T2DM. We conducted the analyses using two models. Covariates in model 1 included: age at interview, total energy intake, total physical activity (MET hours/day), smoking status (never, ex-smoker, or current smoker), alcohol consumption status (never, ex-drinker, or current drinker), education level, occupation, income level, hypertension, and family history of diabetes. In model 2, analyses were conducted with further adjustment for BMI and WHR. Tests for linear trend were performed by entering the categorical variables as continuous parameters in the models. We also investigated a potential interaction between BMI and smoking and the risk of T2DM. The log-likelihood ratio test was used to test the interaction effect of BMI and smoking on T2DM risk. All analyses were performed using SAS software, version 9.2 (SAS Institute, Inc., Cary, North Carolina), and all tests of statistical significance were based on *P* values of <0.05 (two-sided) probability.

## Results

After an average of 5.4 years of follow-up (276 929 person-years), we identified a total of 1304 self-reported T2DM cases; 1227 met our criteria for “confirmed” cases. The incidence rate of T2DM in this sample population was 4.7/1000 person-years. Baseline demographic and other characteristics of the study participants are present in Table 1. A total of 61.8% participants were current smokers and 30.5% were current regular alcohol drinkers. Among current drinkers, 73.4% reported drinking rice wine, 52.9% reported drinking beer, 24.8% reported drinking liquor, and 6.8% reported drinking grape wine regularly (data not shown in tables). A total of 16 916 (32.9%) men reported participation in exercise/sports-related physical activity during leisure time. A total of 71.2% of participants who were working at baseline reported commuting to work by bicycling or walking (data not shown in tables). Participants with T2DM tended to be older and less educated and were more likely to have a higher BMI and WHR. They were also more likely to be less physically active and to have a diagnosis of hypertension and a family history of diabetes. T2DM cases did not differ from the rest of the cohort in income or longest occupation held.

**Table 1:** Demographic characteristics of participants by T2DM status.

		Type 2 Diabetes Mellitus Status			
	All participants	Controls	Cases	*P* value	
	N = 51,464	N = 50,160	N = 1,304		
**Age** (mean ±SD, years)	54.1±9.3	54.1±9.3	55.7±9.3	<0.0001	
**BMI** (mean±SD, Kg/m^2^)	23.6±3.1	23.5±3.0	25.8±2.9	<0.0001	
BMI<25 (%)	68.5	69.3	39.0	<0.0001	
Overweight (25≤BMI<30; %)	29.3	28.6	53.2		
Obese (BMI ≥30; %)	2.3	2.1	7.8		
**WHR** (mean±SD)	0.90±0.06	0.90±0.06	0.93±0.05	<0.0001	
**Family History of Diabetes** (%)	16.5	16.2	27.1	<0.0001	
**Hypertension** (%)	24.5	24.0	43.6	<0.0001	
**Education** (%)				0.03	
None/Elementary	5.6	5.5	6.3		
Middle school	33.7	33.7	36.8		
High school	37.5	37.6	34.3		
College and above	23.2	23.2	22.6		
**Income** (%)				0.40	
<500	13.2	13.2	13.1		
500–999	41.6	41.8	39.7		
1,000–1,999	35.2	35.1	37.3		
>1,999	9.8	9.8	9.9		
**Occupation** (%)				0.15	
Professional	25.3	25.3	26.5		
Clerical	22.0	22.0	23.5		
Manual laborers	52.7	52.8	50.0		
**Currently Working** (at baseline; %)	65.9	66.0	61.9	0.002	
**Energy Intake** (Kcal/day)	1927.4±484.8	1926.9±484.3	1947.5±500.8	0.13	
**LPA Participation** (%)	32.9	32.9	32.8	0.97	
**Total Physical Activity** (mean±SD,METs-h/y d)	8.5±4.9	8.5±4.8	8.1±5.0	0.02	
**Smoking status** (%)				0.03	
Never	29.1	29.1	30.8		
Ex-smoker	9.1	9.1	10.7		
Current smoker	61.8	61.8	58.5		
**Drinking status** (%)				0.001	
Never	66.0	65.9	68.5		
Ex-drinker	3.6	3.5	4.8		
Current drinker	30.5	30.6	26.7		

T-tests were used for continuous variables; chi-square tests were used for categorical variables.

Table 2 presents associations between different types of physical activity and risk of T2DM. We found that total physical activity in METs was associated with reduced risk of T2DM. HRs across quintiles of total METs were 1.00, 0.84, 0.72, 0.66, and 0.65 (*P*-trend <0.0001) in multivariate-adjusted model 1. We found an inverse association between LPA participation and risk of T2DM (HR = 0.85, 95%CI: 0.75–0.96). LPA METs did not appear to be associated with risk of T2DM in this population. DPA was strongly associated with reduced risk of T2DM, HRs for the lowest to highest quintiles were 1.00, 0.83, 0.82, 0.77, and 0.69 (*P*-trend <0.0001) in multivariate-adjusted model 1. CPA was categorized into 3 groups: low, medium, and high according to the MET levels. The HRs were 1.00, 0.86, and 0.66 (*P*-trend <0.0001). The association between physical activity and T2DM was unchanged when further adjustments for BMI and WHR were made (model 2).

**Table 2:** Associations between types of physical activity and T2DM risk.

	No. of diabetescases	HR*	95%CI	HR**	95%CI
**Total METs**					
Q1 (<4.3)	307	1.00		1.00	
Q2 (4.3–<6.5)	272	0.84	0.72–0.99	0.92	0.78–1.08
Q3 (6.5–<8.9)	242	0.72	0.61–0.85	0.80	0.68–0.95
Q4 (8.9–<12.1)	233	0.66	0.55–0.78	0.74	0.62–0.88
Q5 (≥12.1)	250	0.65	0.54–0.77	0.73	0.61–0.87
		P trend<0.0001		P trend<0.0001	
**LPA participation**					
None	876	1.00			
Yes	428	0.85	0.75–0.96	0.86	0.76–0.98
**LPA METs**					
None	876	1.00		1.00	
Low (<1.2)	117	0.79	0.65–0.96	0.80	0.65–0.97
Medium (1.2–3.0)	137	0.87	0.72–1.04	0.89	0.74–1.07
High (≥3.0)	174	0.89	0.75–1.07	0.91	0.76–1.08
		P trend = 0.0763		P trend = 0.1195	
**DPA METs**					
Q1 (<3.3)	296	1.00		1.00	
Q2 (3.3–<5.03)	253	0.83	0.70–0.98	0.88	0.74–1.04
Q3 (5.03–<7.0)	257	0.82	0.69–0.97	0.88	0.74–1.04
Q4 (7.0–<9.8)	251	0.77	0.65–0.91	0.85	0.72–1.01
Q5 (≥9.8)	247	0.69	0.58–0.83	0.75	0.63–0.90
		P trend <0.0001		P trend = 0.0033	
**CPA METs** ***					
Low (<1.1)	427	1.00		1.00	
Medium (1.1–2.67)	219	0.86	0.73–1.01	0.93	0.79–1.10
High (≥2.67)	161	0.66	0.55–0.79	0.74	0.62–0.89
		P trend <0.0001		P trend = 0.0022	

* Model 1: Adjusted for age at interview, energy intake, smoking, alcohol consumption, education level, occupation, income level, hypertension, and family history of diabetes.

** Model 2: As above plus BMI and WHR.

*** Analysis restricted to employed participants.

Smoking more than 20 cigarettes per day and a smoking duration of ≥40 pack-years were associated with increased risk of T2DM (HR = 1.41, 95%CI: 1.13–1.77 for smoking >20 cigarettes/day; HR = 1.39, 95%CI: 1.13–1.71 for ≥40 pack-years of smoking). The associations were still significant after further adjustment for BMI and WHR (Table 3).

**Table 3:** Associations between cigarette smoking and T2DM risk.

	No. of diabetescases	HR*	95%CI	HR**	95%CI
**Smoking status**					
Non-smoker	402	1.00		1.00	
Ex-smoker	139	1.07	0.88–1.30	0.97	0.80–1.18
Current smokers	763	1.06	0.93–1.21	1.06	0.92–1.21
**Number of cigarettes smoked**					
Non-smoker	402	1.00		1.00	
Ex-smoker	139	1.07	0.88–1.30	0.97	0.80–1.18
≤10 cigarettes	251	0.97	0.82–1.14	0.99	0.84–1.17
11–20 cigarettes	405	1.07	0.92–1.25	1.07	0.92–1.24
>20 cigarettes	107	1.41	1.13–1.77	1.25	1.00–1.56
**Pack-years**					
Never smoker	402	1.00		1.00	
Ex-smoker	139	1.08	0.88–1.31	0.97	0.80–1.18
<20	303	0.98	0.84–1.15	1.01	0.86–1.18
20–<40	335	1.03	0.88–1.20	1.02	0.87–1.20
≥40	125	1.39	1.13–1.71	1.28	1.04–1.57

* Model 1: Adjusted for age, energy intake, total physical activity METs, alcohol consumption, education level, occupation, income level, hypertension, and family history of diabetes.

** Model 2: As above plus BMI and WHR.

We conducted an interaction test between smoking and BMI and the risk of T2DM (data not shown in tables). We did not observe an interaction between the number of cigarettes smoked per day and BMI (*P* = 0.059) nor between pack-years and BMI (*P* = 0.1159).

Moderate alcohol consumption (1–3 drinks/day) was associated with reduced risk of T2DM (HR = 0.81, 95%CI: 0.68–0.96) in model 1, whereas occasional (light) and heavy alcohol consumption were not associated with risk of T2DM in this population. The inverse association was mainly observed among wine drinkers. There were no significant associations between beer or liquor consumption and risk of T2DM. Results were similar for model 2 (Table 4).

**Table 4:** Associations between alcohol consumption and T2DM risk*.

	No. of diabetescases	HR**	95% CI	HR***	95%CI
**Alcohol consumption**					
Non-drinker	893	1.00		1.00	
Occasional/light (<1 drink/day)	74	0.88	0.69–1.11	0.88	0.70–1.12
Moderate (1–<3 drinks/day)	172	0.81	0.68–0.96	0.80	0.67–0.94
Heavy (≥3 drinks/day)	102	0.98	0.79–1.21	0.91	0.74–1.13
**Wine**					
None	978	1.00		1.00	
<1 drink/day	116	0.81	0.67–0.99	0.82	0.67–0.99
1–<3 drinks/day	108	0.82	0.67–1.01	0.80	0.65–0.97
≥3 drinks/day	39	1.02	0.74–1.41	0.93	0.67–1.29
**Beer**					
None	1054	1.00		1.00	
<1 drink/day	146	0.95	0.80–1.14	0.98	0.82–1.16
≥1 drink/day	41	0.94	0.68–1.29	0.95	0.69–1.31
**Liquor**					
None	1144	1.00		1.00	
<1 drink/day	25	1.07	0.72–1.59	1.07	0.72–1.59
≥1 drink/day	72	0.98	0.76–1.25	0.93	0.73–1.19

* Ex-drinkers were excluded from analysis.

** Model 1: Adjusted for age, energy intake, physical activity METs, smoking, education level, occupation, income level, hypertension, and family history of diabetes.

** Model 2: As above plus BMI and WHR.

## Discussion

In this prospective cohort study of Chinese men, we found that physical activity was associated with lower risk of T2DM, consistent with the findings of many previous epidemiological investigations of Western populations [14]–[18]. The biological mechanisms underlying this protective effect have been attributed to the ability of physical activity to promote glucose metabolism, control body fatness, and improve insulin sensitivity [19]. While the association between LPA and T2DM is well documented, studies on associations between other types of physical activity and T2DM are scarce. A study of a middle-aged Pakistani population that found stair climbing was inversely associated with the risk of T2DM, whereas household activities and walking were unrelated to risk [20]. A study conducted in Finland showed that CPA reduced the risk of T2DM among men in analyses adjusted for age and study year (*P*-trend = 0.036), although the association disappeared after multivariate-adjustment (*P*-trend = 0.501) [21]. Other studies have evaluated only the combined effect of LPA and other types of physical activity on incidence of T2DM [16], [22].

The physical activity questionnaire in our study was specifically designed to assess a wide range of activities; thus, we were able to investigate associations between different types of physical activity and incidence of T2DM. As expected, the combination of all three types of physical activity had an inverse, dose-response relationship with T2DM. Moreover, we found that CPA and DPA also reduced the risk of T2DM in this population. The lifestyle characteristics of Chinese men differ from those of men in Western countries. In developed, Western countries, people most often commute via automobile and rarely walk or bicycle to work, but have high participation in exercise/sports. In our study population, participants’ regular LPA involvement was low (32.9%). Our study results suggest that physical activity related to daily living and commuting was as effective as leisure-time exercise/sports-related physical activity in reducing the risk of T2DM. We found similar results in the Shanghai Women’s Health Study, the sister study of the SMHS [23].

Smoking is a major cardiovascular risk factor and the leading cause of avoidable death worldwide [24]. After decades of studies, epidemiological evidence has firmly linked cigarette smoking with T2DM risk [25], although most studies have been conducted in Western populations. China is the largest consumer and producer of tobacco in the world. In 2010, there were 301 million smokers in China, most of whom are men (only 2.4% adult women in China smoke) [26], [27]. In our study, 61.8% of men were current smokers, and the smoking rate peaked between age 40 and 60 years (70.7%, data not shown in tables). Of current smokers, 10.6% smoked more than 1 pack of cigarettes a day, and the average number of cigarettes smoked per day was 16. The average number of pack-years smoked was 24. We found that smoking more than 1 pack of cigarettes per day increased the risk of T2DM by 24%, and smoking more than 40 pack-years increased the risk by 28%. The association between smoking and T2DM may be confounded or modified by BMI. Indeed, a Japanese study found that heavy smoking increased the risk for T2DM among obese men, but light smoking reduced risk among lean men [28]. Other studies have shown that smokers have lower body weight compared with non-smokers, and the number of cigarettes smoked has been inversely associated with BMI [29], [30]. We conducted an interaction test between smoking and BMI but found no interaction effects between the number of cigarettes smoked per day and BMI (*P* = 0.059) nor between pack-years and BMI (*P* = 0.1159).

The association between alcohol consumption and incidence of T2DM is less clear. Some studies have shown alcohol to be positively associated with the incidence of T2DM [31], [32], whereas other studies have reported a null association [33], [34] and some have reported a U-shaped or J-shaped relationship between alcohol consumption and the risk of T2DM [35], [36]. In our study, neither occasional, light, nor heavy alcohol consumption was associated with T2DM. However, moderate alcohol intake (1–3 drinks/day) was inversely related to risk of T2DM. This finding is consistent with the effect of moderate alcohol consumption on improving insulin sensitivity [37]–[39]. While the inconsistent results in the literature on alcohol consumption and T2DM may be related to the amount of alcohol consumed, differences in the types of alcoholic beverages commonly consumed in different populations may also play a role. In our study population, about three-fourths of drinkers reported having consumed rice wine, which is an alcoholic beverage brewed from grains and is commonly consumed in China, but rare in Western countries. The number of participants having more than 3 drinks/day of beer or liquor in our population was low, so we categorized beer and liquor consumption into 3 groups: none, <1 drink/day, and ≥1 drinks/day. This level of beer and liquor intake was not associated with T2DM, despite other studies having linked consumption of beer and other types of alcoholic beverages to increased risk of T2DM [40], [41]. More studies are needed to investigate the association between alcohol consumption and T2DM by types of alcohol and the amount consumed.

Our study is based on a large population-based cohort study with high quality exposure data and high recruitment and follow up rates, which minimized selection bias and measurement errors. Extensive information on covariates allowed for us to fully adjust for many socio-demographic factors and other lifestyle cofounders. The major concern for our study is its reliance on self-reported T2DM, since undiagnosed T2DM cases could have been missed. If a healthy lifestyle is related to health consciousness and more frequent physical check-ups, as has been documented in other populations [42], [43], the associations observed in our study could be underestimated. In addition, our study included only middle age men; thus, our results may not be generalizable to women.

In conclusion, we comprehensively evaluated associations between physical activity, smoking, and alcohol consumption and incidence of T2DM in a large population of Chinese men. We found that physical activity and moderate alcohol intake were associated with decreased risk of T2DM, whereas smoking increased risk. Because these lifestyle factors are modifiable, they should receive the highest priority in program development for T2DM control and prevention.
